# An Exciting Case of Acute on Chronic Hypercalcemia in the Setting of Chronic Primary Hyperparathyroidism: A Case Report and Diagnostic Approach

**DOI:** 10.7759/cureus.31202

**Published:** 2022-11-07

**Authors:** Qalb Khan, Cameron Rattray, Avais Masud, Mayurkumar Patel, Malvika Ramchandani

**Affiliations:** 1 Nephrology, Jersey Shore University Medical Center, Neptune, USA; 2 Internal Medicine, St. George's University School of Medicine, St. George, GRD

**Keywords:** diffuse large b-cell lymphoma (dlbcl), calcitriol-mediated hypercalcemia, non-hodgkin lymphomas, persistent primary hyperparathyroidism, hypercalcemia

## Abstract

Acute worsening of hypercalcemia in patients with chronic primary hyperparathyroidism can be challenging, and availability bias may mislead physicians to diagnose worsening primary hyperparathyroidism, especially if the parathyroid hormone is also trending higher. We report a case of stage-IV non-Hodgkin’s lymphoma which presented as acute worsening of hypercalcemia in a patient with chronic primary hyperparathyroidism.

## Introduction

Hypercalcemia is common in patients with chronic primary hyperparathyroidism. However, if they present with acute on chronic hypercalcemia, availability bias can mislead physicians to diagnose worsening primary hyperparathyroidism without any further workup especially if parathyroid hormone (PTH) is also trending higher. In cases of acute worsening of calcium in patients with chronic hyperparathyroidism, additional workup should always be considered to determine other etiologies of hypercalcemia like thiazides diuretics, granulomatous disorders like sarcoidosis, thyrotoxicosis, milk-alkali syndrome, hypercalcemia of malignancy and paraneoplastic hypercalcemia. We report a case of stage-IV non-Hodgkin’s lymphoma which presented as an acute worsening of mild chronic hypercalcemia in a patient with chronic primary hyperthyroidism.

## Case presentation

An 82-year-old man with a past medical history significant for hypertension, coronary artery disease, diabetes mellitus type 2, chronic primary hyperparathyroidism (status-post parathyroidectomy with stable calcium, phosphorous, and PTH and without current pharmacologic intervention), and chronic kidney disease (CKD) stage-III presented to the emergency department (ED) with a 2-day history of generalized weakness, dizziness, constipation, nausea, and non-bilious vomiting, with the remaining review of systems being negative. The patient’s clinical examination was benign except for signs of mild hypovolemia. Initial laboratory studies showed the following abnormal laboratory results (Table 1), elevated serum creatinine of 1.97 mg/dL from a baseline of 1.4-1.5 mg/dL, elevated calcium of 14.9 mg/dL from a baseline of 10.5-10.8 mg/dL for a few years, elevated PTH of 253.4 pg/mL from a baseline in the 160s for a few years, decreased serum albumin of 2.7 g/dL, decreased total protein of 5.1g/dL, and a decreased anion gap of 5 mmol/L.

**Table 1:** Abnormal Laboratory Values Measured H = high; L = low

Abnormal Parameters	Lab Value	Normal Value
Serum Creatinine	1.97 mg/dL (H)	0.61-1.24 mg/dL
Serum Calcium	14.9 mg/dL (H)	8.5 to 10.2 mg/dL
Serum Parathyroid Hormone	253.4 pg/mL (H)	12-88 pg/mL
Serum Albumin	2.7 g/dL (L)	3.5-5.0 g/dL
Serum Total Protein	5.1 g/dL (L)	6.0-8.0 g/dL
Serum Anion Gap	5 mmol/L (L)	6-12 mmol/L
Serum 1,25-dihydroxy-Vitamin D	88 pg/mL (H)	18-72 pg/mL

Upon admission, the patient’s exacerbated hypercalcemia was thought to be from worsening primary hyperparathyroidism and hypovolemia in the setting of nausea, vomiting, decreased oral intake, and hypercalcemia-induced diuresis. Details of treatment are given later below. Given the low albumin levels and low anion gap, serum and urine protein electrophoresis were measured but proved negative. Other secondary hypercalcemia workup showed a normal 25-hydroxy-vitamin D level of 39 pg/mL (normal range: 30-100 pg/mL), a slightly elevated 1,25-dihydroxy-vitamin D (calcitriol) level of 88 pg/mL and a normal parathyroid hormone-related peptide (PTHrP) level of 13 pg/mL (normal range: 11-20 pg/mL) (Table 1).

Ongoing symptoms of abdominal pain, nausea, and vomiting resulted in the patient receiving a computer tomography (CT) of the abdomen and pelvis, which showed abdominal and pelvic lymphadenopathy likely secondary to an inflammatory or neoplastic process. Later, a lymph node biopsy was pursued and revealed fragmented needle-shaped lymphoid tissue with a vaguely nodular pattern. The nodules consisted of large neoplastic cells with oval nuclei, fine chromatin, prominent nucleoli, and a moderate amount of cytoplasm. These cells were positive for CD20, BCL-2, BCL-6, MUM1, and Myc and were negative for CD3, CD10, CD21, and cyclin D1, consistent with diffuse large B-cell lymphoma (DLBCL). The patient subsequently followed hematology and oncology to treat B-cell-originating non-Hodgkin’s lymphoma of the spleen. Acute worsening of hypercalcemia is thought to be due to DLBCL causing elevated levels of calcitriol and hypercalcemia.

In-patient management for acute hypercalcemia

The patient was admitted for a possible exacerbation of primary hyperparathyroidism in the presence of acute on CKD and hypovolemia. Initial treatment included IV fluids and cinacalcet 30 mg oral BID, intramuscular calcitonin 4 units/kg daily, one dose of zoledronate 3 mg infusion, and intravenous normal saline. Calcium levels were closely monitored and trended down, ultimately returning to normal on the patient’s fifth day of admission.

The patient was later treated by hematology for non-Hodgkin’s lymphoma.

## Discussion

The diagnostic approach for hypercalcemia typically begins with the measurement of PTH, which will generally distinguish between the two most common causes of hypercalcemia: primary hyperparathyroidism and malignancy. In primary hyperparathyroidism, PTH may be frankly or inappropriately elevated (as in our patient) with hypercalcemia [1-3]. Additionally, it is typically rare for primary hyperparathyroidism to present with the classic symptoms of hypercalcemia and is often discovered incidentally. Elevated PTH may also be recognized in tertiary hyperparathyroidism and familial hypocalciuric hypercalcemia (FHH). Tertiary hyperparathyroidism is typically associated with PTH increases to maintain normal serum calcium in the setting of vitamin D deficiency and is usually secondary to CKD. FHH is a genetic disorder caused by a mutation in the calcium-sensing receptor gene, CaSR [1].

In patients with hypercalcemia of lymphoma, PTH is suppressed due to an increased production of 1-ɑ-hydroxylase, which stimulates the formation of 1,25-dihydroxy-vitamin D and suppresses PTH. Another mechanism for hypercalcemia of malignancy is the production of PTHrP that induces bone and kidney activity to increase serum calcium without endocrine impacts [4,5].

In our reported case, the clinical picture of worsening hypercalcemia in patients with chronic primary hyperparathyroidism was thought to be due to worsening chronic primary hyperparathyroidism, CKD, and hypovolemia. However, a diagnostic workup showed the acute exacerbation of hypercalcemia was primarily due to hypercalcemia from lymphoma and contributed to some extent by chronic primary hyperparathyroidism. Hence, in patients with chronic hypercalcemia and primary hyperparathyroidism with worsening hypercalcemia, secondary causes should be ruled out despite high PTH.

**Figure 1:**
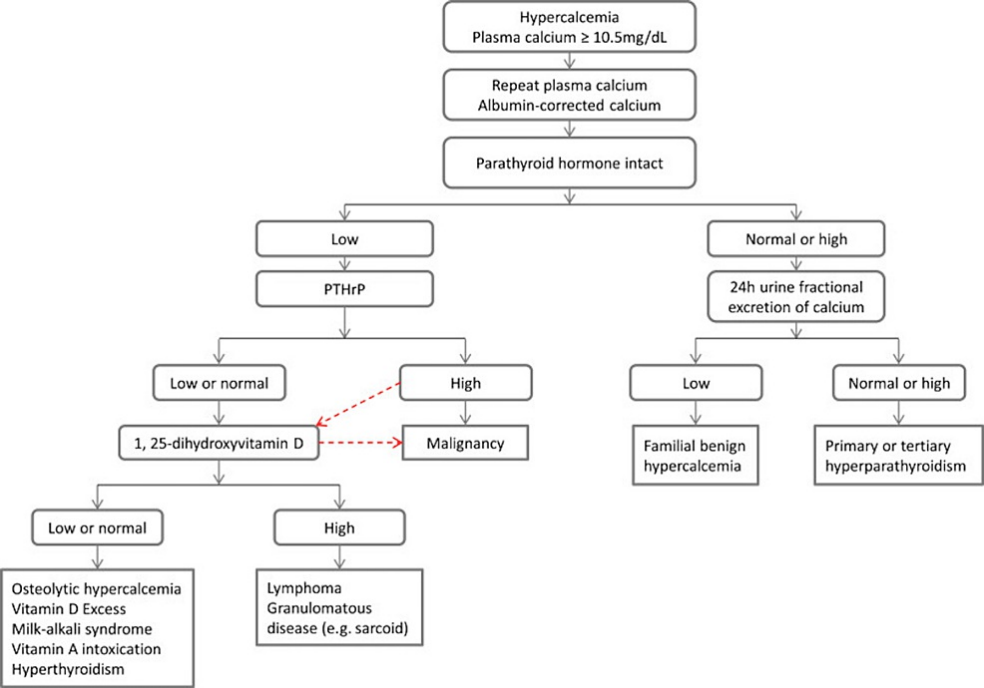
The current diagnostic algorithm of hypercalcemia of malignancy This figure is licensed under a Creative Commons Attribution 4.0 Generic License. No changes were made to the figure. It is attributed to Song et al. Any use of this figure should be attributed to [6].

When utilizing the diagnostic algorithm for hypercalcemia (Figure 1), it is crucial to consider the patient’s presentation, including the absence or presence of B-symptoms [6,7]. In our patient, the only symptoms noted on admission were that of hypercalcemia, and no B-symptoms were acknowledged. Our patient’s presentation was atypical for non-Hodgkin’s lymphoma and further complicated the diagnostic approach to the patient’s hypercalcemia.

Calcitriol-mediated hypercalcemia is the most common cause of hypercalcemia in hematological malignancies and is recognized as a distinct syndrome in Hodgkin’s disease and non-Hodgkin’s lymphoma [8,9]. Patients with malignancy-associated hypercalcemia are usually symptomatic, which adds significant risk to these patients’ morbidity and mortality and may induce oncologic emergencies.

Notably, 85% of calcitriol in plasma is typically bound to vitamin-D-binding protein, the remainder being bound to albumin (15%), and less than 1% exists as a free fraction [9]. In hypoalbuminemia settings, the free calcitriol level may increase, inducing further increases in serum calcium and further exacerbating symptomatic hypercalcemia [10]. Elevated calcitriol and PTHrP within normal limits were demonstrated in our patient, suggesting calcitriol as the primary etiologic culprit for this patient’s malignancy-associated and exacerbated hypercalcemia, which induced his acute symptomatology.

## Conclusions

Hypercalcemia in chronic primary hyperparathyroidism settings is usually an expected laboratory finding. However, in these patients with acute worsening of hypercalcemia, availability bias can mislead physicians to diagnose worsening primary hyperparathyroidism if PTH is trending higher than baseline, as in our reported case. The clinical picture of worsening hypercalcemia in this patient with chronic primary hyperparathyroidism was thought to be due to worsening chronic primary hyperparathyroidism, CKD, and hypovolemia. However, a diagnostic workup showed the patient’s hypercalcemia was primarily due to hypercalcemia from lymphoma and contributed to some extent by chronic primary hyperparathyroidism. Hence, while completing the workup for hypercalcemia, in cases like our patient, physicians should not assume consistently elevated PTH is solely to blame for acute exacerbations of hypercalcemia and treat patients for only primary hyperparathyroidism but should instead evaluate the whole clinical picture and rule in or out a secondary etiology of the acute on chronic hypercalcemia.
